# Quantity of ethanol absorption after excessive hand disinfection using three commercially available hand rubs is minimal and below toxic levels for humans

**DOI:** 10.1186/1471-2334-7-117

**Published:** 2007-10-11

**Authors:** Axel Kramer, Harald Below, Nora Bieber, Guenter Kampf, Cyril D Toma, Nils-Olaf Huebner, Ojan Assadian

**Affiliations:** 1 Institute of Hygiene and Environmental Medicine, Ernst Moritz Arndt University, Walther-Rathenau-Str. 49a, 17489 Greifswald, Germany; 2 BODE Chemie GmbH & Co. KG, Scientific Affairs, Melanchthonstrasse 27, 22525 Hamburg, Germany; 3 Prince Court Medical Centre, 39 Jalan Kia Peng, 50450 Kuala Lumpur, Malaysia; 4 Department of Hygiene and Clinical Microbiology, Medical University of Vienna, Vienna General Hospital, Waehringer Guertel 18-20, 1090 Vienna, Austria

## Abstract

**Background:**

Despite the increasing promotion of alcohol-based hand rubs and the worldwide use of ethanol-based hand rubs in hospitals only few studies have specifically addressed the issue of ethanol absorption when repeatedly applied to human skin. The aim of this study was to assess if ethanol absorption occurs during hygienic and surgical hand disinfection using three different alcohol-based hand-rubs, and to quantify absorption levels in humans.

**Methods:**

Twelve volunteers applied three hand-rubs containing 95% (hand-rub A), 85% (hand-rub B) and 55% ethanol (hand-rub C; all w/w). For hygienic hand disinfection, 4 mL were applied 20 times for 30 s, with 1 minute break between applications. For surgical hand disinfection, 20 mL of each hand rub was applied to hands and arms up to the level of the elbow 10 times for 3 minutes, with a break of 5 minutes between applications. Blood concentrations of ethanol and acetaldehyde were determined immediately prior and up to 90 minutes after application using head space gas chromatography.

**Results:**

The median of absorbed ethanol after hygienic hand disinfection was 1365 mg (A), 630 mg (B), and 358 mg (C). The proportion of absorbed ethanol was 2.3% (A), 1.1% (B), and 0.9% (C). After surgical hand disinfection, the median of absorbed ethanol was 1067 mg (A), 1542 mg (B), and 477 mg (C). The proportion of absorbed ethanol was 0.7% (A), 1.1% (B), and 0.5% (C). The highest median acetaldehyde concentration after 20 hygienic hand disinfections was 0.57 mg/L (hand-rub C, after 30 min), after 10 surgical hand disinfections 3.99 mg/L (hand-rub A, after 20 minutes).

**Conclusion:**

The overall dermal and pulmonary absorption of ethanol was below toxic levels in humans and allows the conclusion that the use of the evaluated ethanol-based hand-rubs is safe.

## Background

The use of alcohol-based hand rubs is a well established method for reducing transient and resident flora on hands [1,2] and there is substantial evidence that hand disinfection reduces the incidence of healthcare-associated infections [3-6]. Most alcohol-based hand rubs contain ethanol, propan-1-ol or propan-2-ol, or a combination of two of these alcohols [7-9]. Alcohol-based hand rubs are mainly used for two purposes: hygienic hand disinfection (Europe) or post-contamination treatment of hands (USA), and surgical hand disinfection (Europe) or pre-operative treatment of hands (USA).

In Europe, use of ethanol for purposes of hygienic hand disinfection has been propagated since the end of the 19^th ^century with studies published by Fuerbringer in 1888 [10] and Ahlfeld in 1895 [11]. In the late fifties Neumann and Walz [12] introduced the propanols to be used for hand disinfection. Today, different formulations with variable concentrations of these alcohols are used with the aim to reduce both the transient and resident flora on hands in order to prevent transmission of nosocomial pathogens in hospitals [1]. The antimicrobial efficacy of ethanol is dependent on its concentration. Lower concentrations of ethanol (≤ 70%) have been described to be significantly less effective than higher concentrations (≥ 75%) [13]. Ethanol at a concentration ranging between 60% and 95% is generally classified to be safe and effective for topical use on hands [14]. Both, the CDC-guideline for hand hygiene [3], and the recently published WHO guideline on hand hygiene in healthcare [15] clearly favour the use of alcohol-based hand rubs in hospitals because other alternatives like antimicrobial soaps have significant disadvantages such as a lower efficacy [1,16], a decreased dermal tolerance [1,17], higher potential for impaired efficacy due to an incorrect performance of the procedure [18], the necessity of a wash basin, and the longer time spent for the procedure [19]. However, despite the increasing promotion of alcohol-based hand rubs and the worldwide use of ethanol-based hand rubs in hospitals only few studies have specifically addressed the issue of ethanol absorption when repeatedly applied to human skin. Generally, it is stated that ethanol is absorbed by human skin in a quantity described as "toxicologically negligible". Yet, this opinion is based on earlier studies, in which the concentration of ethanol in serum was not investigated [20-24], or contradictory results were presented. Two investigators reported that no rise of ethanol concentrations in human serum were detectable, even when excessive ethanol exposure occurred using dressings soaked with 200 ml ethanol for 3 h [25,26]. Yet, this experimental design does not allow drawing valid conclusions for hand hygiene procedures.

In light of the recent WHO recommendations [15] the possibility of ethanol absorption from skin in man is not of trivial nature. Some cultures and religions particularly Islam, categorically prohibit the use of alcohol and regard its use as a sin ('haram') [27]. Ethanol is the principal alcohol found in all alcoholic beverages. In Islam, all intoxicants are haram whether they are in liquid, solid or in any other form regardless of its quantity. Although never investigated, for Muslims alcohol skin absorption and its smell might arguably constitute a perceptive barrier for the use of alcohol-based hand rubs and concerns have been expressed about the potential systemic diffusion of alcohol or its metabolites following dermal absorption or airborne inhalation related to the use of alcohol-based hand rub formulations. As a result, the adoption of alcohol-based formulations as the gold standard for hand hygiene may be unsuitable or inappropriate for some healthcare workers, either because of their reluctance to have contact with alcohol, or because of their concern about alcohol absorption by route of the skin.

Currently available scientific data, elucidating this issue are limited or inconclusive. The aim of this study was therefore to assess if absorption of ethanol does occurs using three different alcohol-based hand rubs for hygienic and surgical hand disinfection, and if so, whether its quantity is minimal or below toxic levels for humans.

## Methods

### Setting

Hand rubs were applied in a room sized 37 m^3 ^with two open windows and an open door. No controlled air exchange occurred during applications. Between applications of hand rubs, volunteers were placed in a second room in which the use of alcohol-based hand rubs was not permitted. Blood samples were collected in a third room.

### Volunteers

All hand rubs were tested on the same 12 volunteers (6 male, 6 female). Inclusion criteria were a minimum age of 18 years and the ability to perform a standardized application according to EN 1500:1997 [28]. Exclusion criteria were defined as follows: visible skin lesions on hands or arms, skin disease, alimentary intake of ethanol in any form within 24 h before the beginning of an experiment, diabetes mellitus, pregnancy or lactation, and participation in a clinical trial 30 days prior to start of this study. Written consent was obtained from all volunteers. The study was approved by the Ethics Committee of the Board of Physicians Mecklenburg-West Pomerania at the University of Greifswald.

### Hand rubs

Three blinded ethanol-based hand rubs were tested: hand rub A (Sterillium^® ^Virugard, 95% w/w ethanol, density 0.789 g/mL, Bode Chemie GmbH, Hamburg, Germany); hand rub B (Sterillium^® ^Gel, 85% w/w ethanol, density 0.826 g/mL, Bode Chemie GmbH, Hamburg, Germany); and hand rub C (Manorapid Synergy^®^, 55% w/w ethanol in combination with 10% w/w propan-1-ol, ethanol density 0.900 g/mL, Antiseptica GmbH, Pulheim, Germany).

### Estimation of the application frequency

Hygienic hand disinfection is performed after a proven or anticipated contamination of hands [3]. Although the compliance rate in hand hygiene on average only amounts to 50%, it can be safely assumed that approximately on average 20 hygienic hand disinfections are carried out per healthcare worker per shift [19]. This number is certainly variable depending on the nature of clinical activity, the clinical setting, or the impact of training programs [6]. The risk of contamination of the hands of healthcare workers and the susceptibility of patients for acquiring a healthcare-associated infections is, for example, much lower in a psychiatric setting than in intensive care units. Under practical conditions the procedure of hand disinfection averages between 6 – 24 s and normally does not reach the recommended 30 s [6]. The exposure of a healthcare worker to ethanol in a "real life" situation can therefore only be estimated based on the number of hygienic hand disinfections which is likely to be on average 5–6 minutes per healthcare worker and shift [29].

Surgical hand disinfection is carried out before each surgical procedure with a compliance of nearly 100%. It can be assumed that surgical healthcare workers perform an average of 4 surgical hand disinfections per day. The contact time of ethanol with human skin will be approximately 3 minutes per surgical hand disinfection for most preparations [30]. Therefore the exposure to ethanol is likely to be on average 12 minutes per healthcare worker per shift.

### Application of hand rubs

Immediately prior to the initiation of the experiments, the hands were washed with non-medicated neutral pH soap and dried thoroughly. For hygienic and surgical hand disinfection each hand rub was tested individually on one of three consecutive days of evaluation. For each application 4 mL of a hand rub were applied in the test room to both hands and rubbed in for 30 s according to the standard rub-in procedure described in the European norm EN 1500:1997 [28]. After a waiting time of 1 minute outside the test room, the procedure was repeated. A total of 20 hygienic hand disinfections were performed, resulting in a total exposure time with each hand rub of 10 minutes over a period of 30 minutes.

Surgical hand disinfection experiments started 7 days after the hygienic hand disinfection experiments. Four mL of the hand rub were applied to the hands and rubbed on hands and forearms. This procedure was repeated five times with the aim to keep hands and forearms covered with the hand rub for the recommended application time of 3 minutes [31]. After a waiting time of 5 minutes outside the test room the procedure was repeated. A total of 10 surgical hand disinfections were performed resulting in a total exposure time with each hand rub of 30 minutes over a period of 80 minutes. At the end of each test day a dermatological protective hand cream was applied to the treated skin areas.

### Blood sampling

Prior to sampling, the skin was disinfected with an alcohol-free skin antiseptic (alcohol-free povidone-iodine, 1 minute). In order to determine the ethanol concentration before the first application of a day (baseline) and 2.5, 5, 10, 20, 30, 60 and 90 minutes after the last hygienic hand disinfection or 5, 10, 20, 30, 60 and 90 minutes after the last surgical hand disinfection, respectively, 5 mL of venous blood were drawn through a peripheral intravascular catheter (BD Inside-W™, 18 GA, Becton Dickinson Sandy, Utah, USA). Only for hand rub C, an additional sample was taken 120 minutes after the last surgical hand disinfection. Blood samples were stored before analysis at 4°C for up to 12 h.

### Analysis of ethanol and acetaldehyde concentration

The measurement quantification of ethanol and acetaldehyde concentrations in peripheral blood was performed using gas chromatography in a modification of the method described by Roemhild et al. [32]. This technique uses head-space injection (CombiPal-Autosampler, CTC Analytics) with flame-ionization detection (Gas chromatograph 5890 series II, Hewlett Packard). 1 mL sample or 1 mL standard and 0.5 g glowed Na_2_SO_4 _were filled in 1.5 mL head space vials and incubated 45 minutes at 75°C. Then, 2.5 mL were injected (time interval 0.5 minutes). A DB 624 column (60 m × 0.32 mm × 1.8 μm; J&W Scientific, Folsom, USA) was used for separation. The conditions of the chromatography were 150°C injector temperature, 250°C detector temperature, column temperature program 40°C (8 minutes), 3°C/minutes to 120°C (0 minutes), and 30°C/minutes to 230°C (5 minutes). Nitrogen (5.0) served as carrier gas with 1.45 mL/minute (21.9 cm/s).

In each case, calibration was performed according to the method of the external standard, with three calibration points. Both commercially available standards (Medidrug BGS S, Level 1–3, Medichem) as well as self-made standards were utilized. The latter were used if the sample concentration did not fall within the calibration level (e.g., ethanol) or substances were quantified which are not included in the commercially available standards (e.g. acetaldehyde). These calibration standards were produced by weight of the contents of original substances followed by dilution on calibration level. The content of the self-made standards were cross checked with those of commercially standards by gas chromatographic measurements.

Characteristic analytical data for the procedure used in the determination of acetaldehyde is 0.07 mg/mL and for ethanol 0.14 mg/mL (detection limit), acetaldehyde 0.15 mg/mL and ethanol 0.28 mg/mL (determination limit), and acetaldehyde 0.29 mg/mL and ethanol 0.34 mg/mL (recording limit) (straight line calibration method in accordance with German Standard Organization (DIN 32645) [33].

Legal limits on blood alcohol for drivers of vehicles are typically 500–1000 mg/L. The WHO's recommendation for ethanol is a maximum of 7000 mg per day.

### Data calculation and statistical analysis

For each time point, the median ethanol and acetaldehyde concentration together with its 95% confidence intervals (95% CI) was calculated. If ethanol or acetaldehyde concentrations were below the detection limit, 50% of the value of the detection limit was assumed. Hence, for values below the detection limits, for ethanol a concentration of 0.07 mg/L, and for acetaldehyde a concentration of 0.035 mg/L were assumed.

The amount of absorbed ethanol was determined for each volunteer, each hand rub and mode of application. In order to control for the difference of ethanol absorption between males and females the formula described by Wittmann et al. [34] was applied: Absorbed amount (mg) = body mass (kg) × r × maximum serum level (mg/L), where r is 0.7 for males, and 0.6 for females, respectively.

The proportion of absorbed ethanol was determined for each hand rub and type of application as the ratio of the median absorbed amount and the amount of ethanol initially applied.

Results were analyzed using Epi-Info 2002 (Epi-Info 2002 software package, Centers for Disease Control and Prevention, GA, Atlanta). Continuous variables were analyzed to evaluate normality of distribution. For non-normal distribution, variables were expressed as median together with the 95% confidence intervals (95% CI). Based on the null hypothesis of no differences in the median ethanol or acetaldehyde concentrations between baseline and post-application, P-values were calculated using the Wilcoxon rank sum test. All tests of significance were 2-tailed; P values of = .01 were considered significant.

## Results

### Baseline values

In 79.2% of base-line samples (57 of 72), the baseline ethanol concentration was below the limit of detection. The median ethanol concentration was 0.07 mg/L (0.06–0.08 mg/L). The highest baseline ethanol concentration was 1.7 mg/L. For acetaldehyde, 5.5% of the baseline values (4 of 72) were below the limit of detection. The median acetaldehyde concentration was 0.20 mg/L (0.18–0.22 mg/L). The highest baseline acetaldehyde concentration was 1.95 mg/L.

### Hygienic hand disinfection

#### Exposure

During 20 hygienic hand disinfections within a period of 30 minutes and a total contact time of 10 minutes, volunteers were exposed to a total of 80 mL of hand rub corresponding to an ethanol exposure of 60.0 g (hand rub A), 56.2 g ethanol (hand rub B), and 39.6 g ethanol (hand rub C), respectively.

#### Absorption

**Figure 1:**
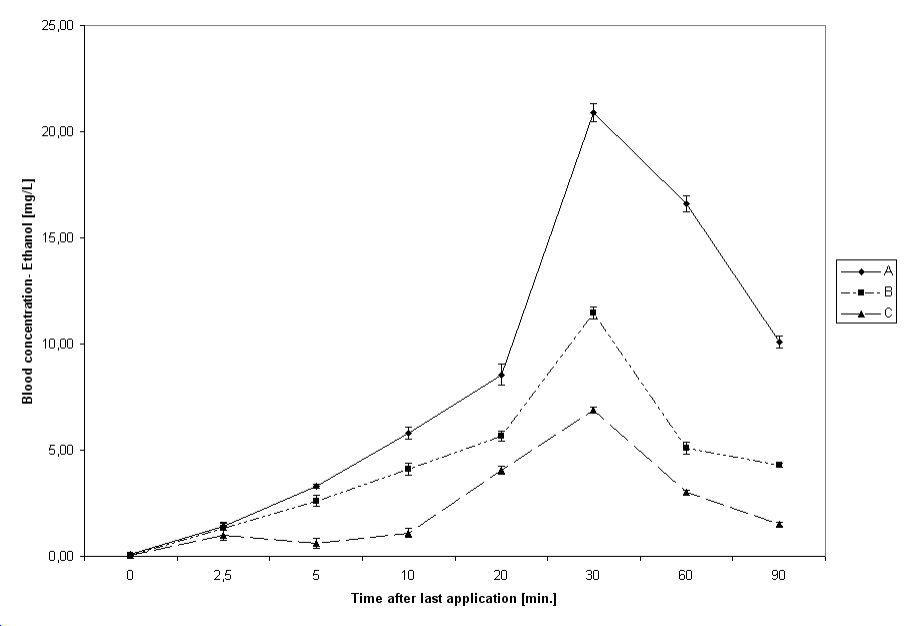
Kinetic of ethanol absorption after hygienic hand disinfection. Bars represent 95% CIs.

After the last application, the median ethanol concentration in peripheral blood increased gradually and peaked after 30 minutes for all hand rubs (Table 1). The highest median concentration found with hand rub A was 20.95 mg/L (equivalent to 0.02‰ ethanol), with hand rub B 11.45 mg/L (equivalent to 0.011‰ ethanol), and 6.90 mg/L with hand rub C (equivalent to 0.007‰ ethanol). After 30 minutes, ethanol concentration gradually decreased for all hand rubs. There was, however, a difference in the absorption kinetics between the tested hand rubs. While for hand rub B (P = 0.003) and C (P = 0.004), the median ethanol concentration started to be statistically significant to the baseline concentration only after 20 minutes, for hand rub A the difference started to be significant after 5 minutes (P = 0.008). (Figure 1)

**Table 1:** Blood concentration of ethanol and acetaldehyde (mg/L) before and after 20 hygienic hand disinfections

Hand rub	Substance	Before first application	Time after last application						
			
			2.5 min	5 min	10 min	20 min	30 min	60 min	90 min
A	Ethanol	0.11 (0.09–0.12)	1.40 (01.22–1.58)	3.30 (3.22–3.38)	5.80 (5.52–6.08)	8.55 (8.06–9.04)	20.95 (20.46–21.34)	16.60 (16.21–16.99)	10.10 (9.83–10.37)
	Acetaldehyde	0.10 (0.01–0.10)	0.20 (0.19–0.21)	0.25 (0.24–0.26)	0.20 (0.18–0.22)	0.30 (0.29–0.31)	0.50 (0.47–0.54)	0.40 (0.39–0.41)	0.20 (0.19–0.21)
B	Ethanol	0.07 (0.06–0.08)	1.30 (1.01–1.59)	2.60 (2.34–2.86)	4.10 (3.79–4.40)	5.65 (5.42–5.88)	11.45 (11.17–11.73)	5.10 (4.83–5.37)	4.30 (4.21–4.32)
	Acetaldehyde	0.10 (0.10–0.10)	0.50 (0.47–0.51)	0.35 (0.34–0.36)	0.30 (0.28–0.32)	0.40 (0.38–0.42)	0.40 (0.39–0.41)	0.40 (0.39–0.41)	0.30 (0.29–0.31)
C	Ethanol	0.07 (0.05–0.08)	1.00 (0.76–1.24)	0.60 (0.37–0.82)	1.10 (0.89–1.31)	4.05 (3.85–4.25)	6.90 (6.76–7.04)	3.00 (2.87–3.13)	1.50 (1.42–1.58)
	Acetaldehyde	0.10 (0.10–0.10)	0.40 (0.39–0.41)	0.30 (0.29–0.31)	0.50 (0.49–0.51)	0.40 (0.38–0.42)	0.60 (0.59–0.61)	0.45 (0.44–0.46)	0.30 (0.28–0.32)

Hand rub A (95% ethanol), hand rub B (85% ethanol), hand rub C (55% ethanol); Median with 95% CI (brackets) from 12 volunteers; total exposure of 60 mL hand rub in an open room of 37 m^3 ^with a total contact time of 10 minutes.

The amount of absorbed ethanol was 1365 mg with hand rub A, 630 mg with hand rub B, and 358 mg with hand rub C. Based on the total amount of applied ethanol with each hand rub, the proportion of absorbed ethanol was 2.3% for hand rub A, 1.1% for hand rub B, and 0.9% for hand rub C.

### Surgical hand disinfection

#### Exposure

During 10 surgical hand disinfections within a period of 80 minutes and a contact time of 30 minutes, volunteers were exposed to a total of 200 mL of hand rub corresponding to a total ethanol exposure of 149.9 g (hand rub A), 140.0 g ethanol (hand rub B), and 99.0 g ethanol (hand rub C), respectively.

#### Absorption

**Figure 2:**
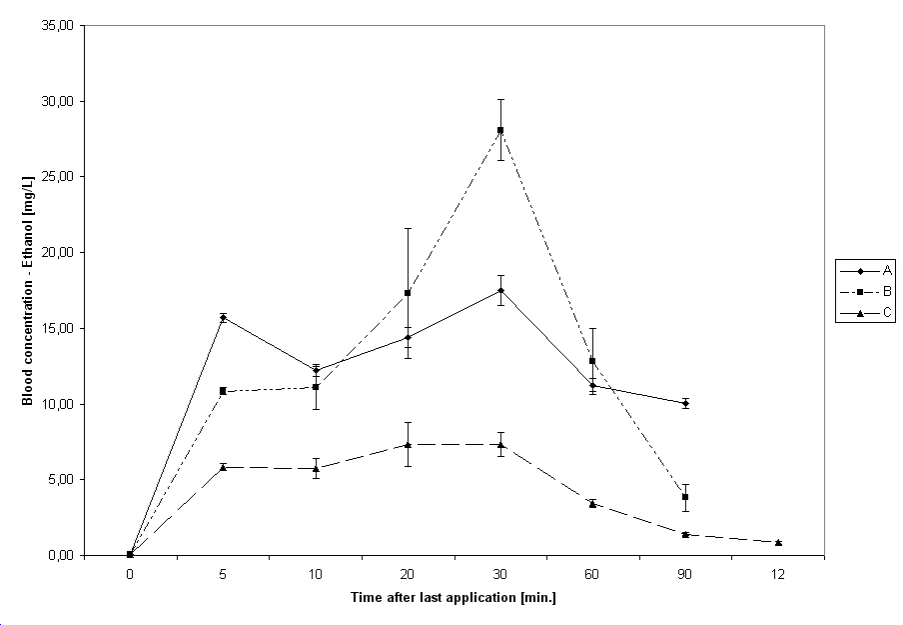
Kinetic of ethanol absorption after surgical hand disinfection. Bars represent 95% CIs.

The highest median ethanol concentration was found with two hand rubs 30 minutes after the last application, but with hand rub C 20 minutes thereafter (Table 2). The maximum observed median ethanol concentration was 17.50 mg/L with hand rub A (equivalent to 0.017‰ ethanol), 30.10 mg/L with hand rub B (equivalent to 0.029‰ ethanol), and 8.80 mg/L with hand rub C (equivalent to 0.008‰ ethanol). For all hand rubs, the median ethanol concentration reached statistical significance to the baseline concentration 5 minutes after the last application (hand rub A, P < 0.001; hand rub B, P < 0.001; hand rub C, P = 0.004). (Figure 2)

**Table 2:** Blood concentration of ethanol and acetaldehyde (mg/L) before and after 10 surgical hand disinfections

Hand rub	Substance	Before first application	Time after last application						
			
			5 min	10 min	20 min	30 min	60 min	90 min	120 min
A	Ethanol	0.07 (0.07–0.07)	15.70 (15.39–16.01)	12.20 (11.81–12.60)	14.40 (13.72–15.08)	17.50 (16.49–18.51)	11.25 (10.84–11.66)	10.06 (9.71–10.38)	Not done
	Acetaldehyde	0.80 (0.78–0.82)	2.70 (2.64–2.76)	3.00 (2.95–3.06)	4.00 (3.90–4.09)	3.55 (3.46–3.64)	2.60 (2.46–2.74)	2.55 (2.44–2.66)	Not done
B	Ethanol	0.07 (0.07–0.07)	11.10 (10.85–11.35)	12.50 (11.09–13.91)	21.60 (17.30–25.89)	30.10 (28.09–32.11)	15.00 (12.81–17.19)	4.70 (3.80–5.60)	Not done
	Acetaldehyde	0.55 (0.54–0.56)	2.30 (2.25–2.36)	2.20 (2.13–2.27)	2.30 (2.21–2.31)	3.30 (3.24–3.36)	2.50 (2.44–2.56)	1.30 (1.24–1.36)	Not done
C	Ethanol	0.07 (0.07–0.07)	6.10 (5.84–6.38)	6.40 (5.74–7.06)	8.80 (7.34–10.26)	8.15 (7.34–8.96)	3.70 (3.44–3.96)	1.50 (1.40–1.59)	0.90 (0.83–0.97)
	Acetaldehyde	0.40 (0.39–0.41)	1.25 (1.21–1.29)	1.50 (1.48–1.53)	1.70 (1.67–1.73)	0.90 (0.86–0.94)	0.70 (0.66–0.74)	1.00 (0.97–1.03)	1.00 (0.98–1.02)

Hand rub A (95% ethanol), hand rub B (85% ethanol), hand rub C (55% ethanol); Median with 95% CI (brackets) from 12 volunteers; total exposure of 200 mL hand rub in an open room of 37 m^3 ^with a total contact time of 30 minutes.

The amount of absorbed ethanol was 1067 mg with hand rub A, 1542 mg with hand rub B, and 477 mg with hand rub C. Based on the total amount of applied ethanol with each hand rub, the proportion of absorbed ethanol was 0.7% for hand rub A, 1.1% for hand rub B, and 0.5% for hand rub C.

### Metabolism

**Figure 3:**
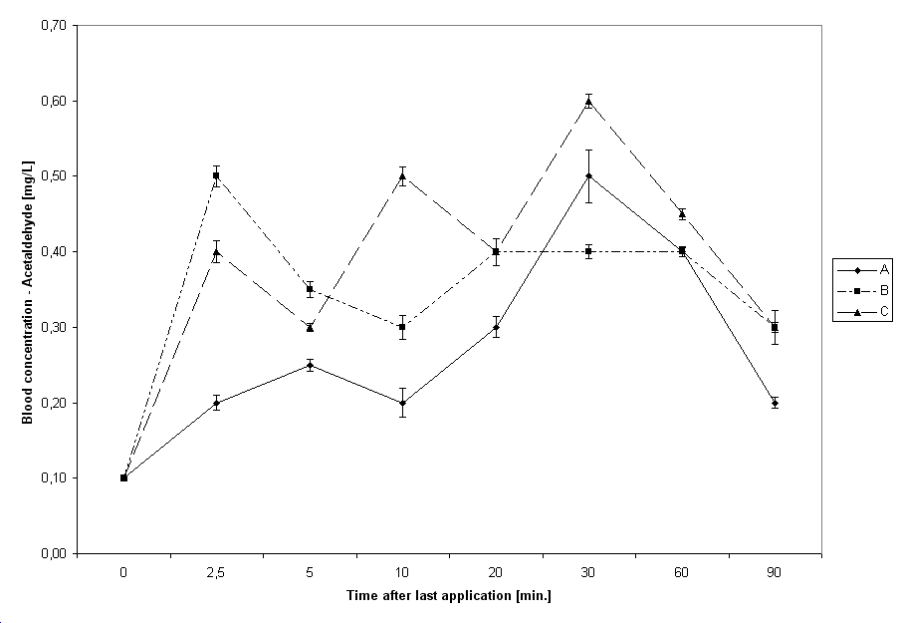
Kinetic of acetaldehyde absorption after hygienic hand disinfection. Bars represent 95% CIs.

**Figure 4:**
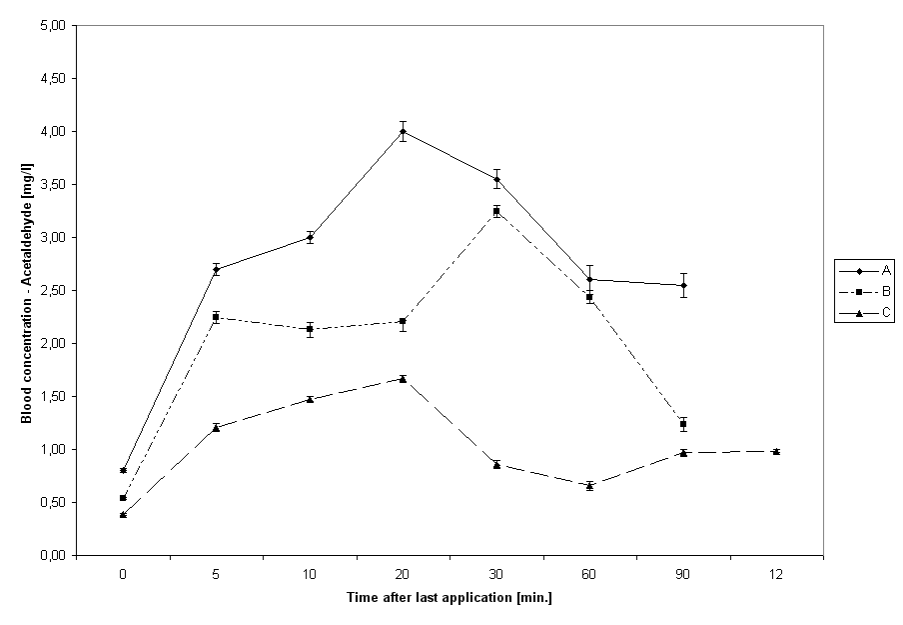
Kinetic of acetaldehyde absorption after surgical hand disinfection. Bars represent 95% CIs.

The highest median acetaldehyde concentrations after 20 hygienic hand disinfections were 0.57 mg/L (hand rub C, after 30 minutes), after 10 surgical hand disinfections 3.99 mg/L (hand rub A, after 20 minutes). After 30 to 60 minutes, however, levels of acetaldehyde decreased gradually (Figures 3 and 4).

## Discussion

Alcohol abuse is a significant medical and social problem. At sufficiently high doses, ethanol, the active ingredient of alcoholic beverages, and others can cause both short-term (such as inebriation) and long-term (such as cirrhosis of the liver) toxic effects in humans. Thus, concern has been raised about the possible health consequences of using ethanol for alcoholic hand rubs. Since the intrinsic toxic effects of ethanol require its entry into the bloodstream, we evaluated ethanol blood concentrations using 3 different ethanol-based hand rubs.

The median baseline values of ethanol (< 0.07 mg/L) and acetaldehyde (0.20 mg/L) indicated ethanol abstinence of volunteers before the initiation of the experiments, since all median observed baseline values were below the maximum physiological level of 0.32 mg/L for ethanol and 0.31 mg/L for acetaldehyde [34]. However, individual baseline ethanol concentrations ranged from non-detectable concentrations to a maximum of 1.70 mg/L. This is not unexpected since ethanol is produced through fermentation by fungi and other intestinal microorganisms, and is found at low levels in the blood and exhalation of individuals otherwise abstinent [35]. The individual blood levels determined in our study at baseline vary to some extent due to individual factors influencing the production, absorption and metabolism of ethanol such as activity of alcohol dehydrogenase, alimentation and gender [36,37].

We were able to demonstrate that following excessive hygienic or surgical hand disinfection only 0.5% to 2.3% of the applied ethanol is absorbed. This excessive exposure, however, will rarely occur in clinical practice. Albeit that, we were compelled to chose this particular experimental design since the literature does not offer data on exact absorption rates after hand disinfection.

Our findings are important to have confidence in the safe use of ethanol-based hand rubs. Blood ethanol levels that result in diminished fine motor coordination range around 200 – 500 mg/L and impaired judgement around 500–1000 mg/L [38]. If a surgeon carries out three surgical hand disinfection with hand rub A (containing the highest concentration of ethanol) over 6 hours (one hand disinfection every two hours) using e.g. a total of 20 mL hand rub, he will be exposed to 15.1 g ethanol every two hours. According to our results, approximately 0.7% of the applied ethanol will be absorbed, equivalent to 106 mg ethanol. Assuming 70 kg body weight and 40.6 L total body water for an average man, or 60 kg body weight and 28.8 L total body water for an average woman, the systemic availability of ethanol after this surgical hand disinfection will be 2.61 mg/L in a man, or 3.68 mg/L in a woman, respectively. These findings are in line with results reported in a recent study by Miller et al. [39] where five subjects applied repeatedly (50 times over 4 hours) 5 mL of an ethanol based hand rub (62% denatured ethyl alcohol) to both hands and rubbed until dry. The authors reported that blood ethanol level upon completion of the applications of the ethanol/based hand rub was less than 5 mg/dL in all 5 study participants.

In comparison, it should be pointed out that a single alcoholic drink contains about 12 g of ethanol [40], corresponds to a dose of 170 mg/kg for a 70 kg adult, and produces a peak blood ethanol concentration of 250 mg/L. Fruit juices may contain up to 3 g ethanol per L [41], and an apple juice may well contain 1 g ethanol per 500 mL. Assuming a resorption rate of 90%, drinking half a litre of apple juice will result in a concentration of 0.17‰ ethanol in a 75 kg man or 0.25‰ ethanol in a 60 kg woman [42].

A hand rub must be safe and effective. Pertaining to safety, we are confident to conclude that under clinical conditions the use of ethanol-based hand rubs does not lead to intoxicating levels of alcohol in the peripheral blood. The efficacy of alcohol-based hand rubs, however, depends on the concentration of alcohol. For patients safety it is therefore a first and foremost prerogative to ensure the efficacy of a hand rub. In light of our results clearly showing that ethanol absorption corresponds to exposure dose and time, it is tempting to speculate that the theoretical risk of systemic toxicity for health care workers could be further minimized by shortening the application time of ethanol in surgical hand disinfection to a minimum time necessary for the alcohol to achieve the required efficacy. This might help to reduce the very small risk of systemic toxicity for the healthcare worker even further. Especially for surgical hand disinfection recent data indicates the possibility of reducing the current recommendation of 3 minutes application time since it was shown that an application time of 1.5 minutes was equally as effective as 2 or 3 minutes [43]. However, so far this has only been shown with a propanol-based hand rub [43,44]. Furthermore, it has been reported that in consecutive surgical procedures of less than 60 minutes duration a 1 minute application may be sufficient to ensure adequate efficacy [45].

Our study has several limitations. We did not take into consideration that the average rate of metabolism for ethanol is 150 mg/L within 1 h or 0.15‰/h, equivalent to 12.5 mg/L within 5 minutes [46]. However, based on this rate of metabolism and an application with longer intervals, the true ethanol blood concentrations will be lower than those calculated in our experimental model. Yet, our setting does not allow predictions about the potential ethanol kinetics for a cumulative absorption over multiple days or weeks of use. Also, our test model did not distinguish between dermal and pulmonary absorption. Prediction of blood ethanol concentration following exposure to ethanol vapours must consider the concentration of ethanol in air, the duration of exposure, breathing rate, absorption of ethanol across the lungs, and the physiological elimination rate of ethanol. The absorption of ethanol across the lungs and the physiological elimination of ethanol are the only two factors more or less constant. In humans, it has been demonstrated that 55% to 60% of inhaled vapours are absorbed into the bloodstream [47]. The clearance rate of ethanol from the blood is about 150 mg/L/hr [48] but may be as high as 230 mg/L/hr [49]. These rates correspond to elimination of 83 mg/kg/hr to 127 mg/kg/hr, or about 6 to 9 g of ethanol per hour for an average adult. However, these considerations are only of academic, but not of practical relevance, since healthcare workers rarely use ventilation masks when applying alcohol-based hand rubs. As in practice healthcare workers also will be exposure to alcohol vapours, we considered the experimental design of this study to be closer to clinical reality. Indeed, some of the observed absorption is certainly due to pulmonary uptake. If for example 200 mL of hand rub A is applied within 80 minutes, a total of 150.1 g ethanol will evaporate into the air. If no air exchange takes place, this will result in an ethanol saturation of 4.1 g/m^3 ^air, which is approximately two times above the maximum occupational exposure concentration of 1.9 g/m^3^. Since both windows and the door of the test room were open, air exchange took place. Nevertheless, it can not be ruled out that some of the ethanol in blood was taken up by respiration.

Although this study provided answers to some hitherto unsolved questions, it can not answer if the use of ethanol-based hand rubs is acceptable for those individuals in which religion or culture prohibits alcohol, i.e. Muslims. Indeed, the data clearly show that after hand disinfection using ethanol-based hand rubs absorption – although non-intoxicating and safe for human level – does occur. This has at least two implications. For Muslims, any substance or process leading to a disconnection from a state of awareness or consciousness is 'haram'. We were able to show that consciousness definitively can not be altered by using different ethanol-based hand rubs. However, this still does not mean that their use is 'halal'. Some Muslims believe that if something taken in a large quantity acts as an intoxicant, then it is 'haram' to even take in a small quantity of that. Yet, others do not share this view. Alcohols can either be 'khamr' or 'non-khamr'. 'Khamr' alcohols can be said to be alcohol derived from dates and grapes while 'non-khamr' alcohols are not derived from any of these two. The ruling regarding 'khamr' is that even the most minuscule amount of it is 'haram', regardless of whether it intoxicates or not, while the ruling considering other alcohols is that only that amount is 'haram' which intoxicates. A small amount which does not cause intoxication is not 'haram'. The only condition is that it must not be drunk for amusement and pastime. If it is used to gain strength, to digest the food, or for medical reasons then it is permissible as long as it does not intoxicate. However, since ethanol is classified as 'khamr', its use could be regarded 'haram', regardless if it could intoxicate or not.

Because this matter could potentially impede the worldwide use of ethanol-based hand rubs, particularly in predominantly Islamic regions, it demands attention and clarification. Yet, at many Saudi Arabian hospitals, use of alcohol-based hand rub has been permitted since 2003, and no difficulties or reluctance to adopt these formulations have been encountered [27]. Moreover, there is an encouraging acceptance in Muslim countries indicating that the use of alcohol-based hands is acceptable to most Muslim health-care workers.

## Conclusion

The overall dermal and pulmonary absorption of ethanol is below toxic levels in humans and allows the conclusion that the use of the evaluated ethanol-based hand-rubs is safe.

## Competing interests

Prof. Dr. Kramer received research funding for the University of Greifswald from Bode Chemie GmbH & Co. KG, Hamburg, Germany, and from Antiseptica GmbH, Pulheim, Germany. PD Dr. Günter Kampf is a paid employee of Bode Chemie GmbH & Co. KG, Hamburg, Germany. Prof. Dr. Assadian, Dr. Huebner, Dr. Bieber, Dr. Toma and Dr. Below have no competing interests.

## Authors' contributions

AK had the idea for the study and planned and supervised the experiments, as well drafted the manuscript and analyzed and interpreted the data. HB participated in the technical design of the study and performed the measurements of ethanol and acetaldehyde; also, he analyzed and interpreted the data. NB and N-OH assisted HB and helped drafting the material and method section of the paper. GK participated in the study's design and coordination, helped to draft the manuscript, and analyzed and interpreted the data. CT advised on issues pertaining to Islamic culture and wrote the respective parts in the discussion section. OA participated in the study design and coordinated to draft and writing the manuscript; he also conducted the statistical analysis of the results and analyzed and interpreted the data. All authors have been involved in drafting the manuscript or revising it critically for important intellectual content and have read and approved the final manuscript.

## Pre-publication history

The pre-publication history for this paper can be accessed here:


